# Role of previous systemic antibiotic therapy on the probability of recurrence after an initial episode of *Clostridioides difficile* infection treated with vancomycin

**DOI:** 10.1093/jacamr/dlad033

**Published:** 2023-03-23

**Authors:** Nicolás Merchante, Rocío Herrero, María Dolores Valverde-Fredet, Miguel Rodríguez-Fernández, Héctor Pinargote, Francisco J Martínez-Marcos, Concepción Gil-Anguita, María García-López, María Tasias Pitarch, Vicente Abril López De Medrano, Miguel Nicolás Navarrete Lorite, Cristina Gómez-Ayerbe, Eva León, Pilar González-De La Aleja, Ana Ruiz Castillo, Ana I Aller, Juan Carlos Rodríguez, Julia Ternero Fonseca, Juan E Corzo, Alberto Naranjo Pérez, Marta Trigo-Rodríguez, Esperanza Merino

**Affiliations:** Unidad Clínica de Enfermedades Infecciosas y Microbiología, Hospital Universitario de Valme, Instituto de Biomedicina de Sevilla (IBiS), Universidad de Sevilla, Avenida de Bellavista s/n, 41014, Sevilla, Spain; Unidad Clínica de Enfermedades Infecciosas y Microbiología, Hospital Universitario de Valme, Instituto de Biomedicina de Sevilla (IBiS), Universidad de Sevilla, Avenida de Bellavista s/n, 41014, Sevilla, Spain; Unidad Clínica de Enfermedades Infecciosas y Microbiología, Hospital Universitario de Valme, Instituto de Biomedicina de Sevilla (IBiS), Universidad de Sevilla, Avenida de Bellavista s/n, 41014, Sevilla, Spain; Unidad Clínica de Enfermedades Infecciosas y Microbiología, Hospital Universitario de Valme, Instituto de Biomedicina de Sevilla (IBiS), Universidad de Sevilla, Avenida de Bellavista s/n, 41014, Sevilla, Spain; Unidad de Enfermedades Infecciosas, Hospital General Universitario Dr. Balmis, Instituto de Investigación Biomédica de Alicante (ISABIAL), Alicante, Spain; Unidad de Enfermedades Infecciosa, Hospital Universitario Juan Ramón Jiménez, Huelva, Spain; Unidad de Enfermedades Infecciosas, Servicio de Medicina Interna, Hospital Marina Baixa, Villajoyosa, Spain; Servico de Medicina Interna, Hospital Vega Baja, Orihuela, Spain; Unidad de Enfermedades Infecciosas, Hospital Universitario y Politécnico La Fe, Valencia, Spain; Servicio de Enfermedades Infecciosas, Consorcio Hospitalario General Universitario de Valencia, Valencia, Spain; Unidad Clínica de Enfermedades Infecciosas, Hospital Universitario Virgen Macarena. Departamento de Medicina, Universidad de Sevilla. Instituto de Biomedicina de Sevilla (IBiS)/CSIC, Sevilla, Spain; CIBER de Enfermedades Infecciosas (CIBERINFEC), Instituto de Salud Carlos III, Madrid, Spain; Unidad de Enfermedades Infecciosas, Hospital Clínico Universitario Virgen de la Victoria, Málaga, Spain; Unidad Clínica de Enfermedades Infecciosas y Microbiología, Hospital Universitario de Valme, Instituto de Biomedicina de Sevilla (IBiS), Universidad de Sevilla, Avenida de Bellavista s/n, 41014, Sevilla, Spain; Unidad de Enfermedades Infecciosas, Hospital General Universitario Dr. Balmis, Instituto de Investigación Biomédica de Alicante (ISABIAL), Alicante, Spain; Servicio Microbiología, Hospital Universitario Juan Ramón Jiménez, Huelva, Spain; Unidad Clínica de Enfermedades Infecciosas y Microbiología, Hospital Universitario de Valme, Instituto de Biomedicina de Sevilla (IBiS), Universidad de Sevilla, Avenida de Bellavista s/n, 41014, Sevilla, Spain; Servicio Microbiología, Hospital General Universitario de Alicante, Instituto de Investigación Biomédica de Alicante (ISABIAL), Alicante, Spain; Servicio de Aparato Digestivo, Hospital Universitario Juan Ramón Jiménez, Huelva, Spain; Unidad Clínica de Enfermedades Infecciosas y Microbiología, Hospital Universitario de Valme, Instituto de Biomedicina de Sevilla (IBiS), Universidad de Sevilla, Avenida de Bellavista s/n, 41014, Sevilla, Spain; Servicio de Aparato Digestivo, Hospital Universitario Juan Ramón Jiménez, Huelva, Spain; Unidad Clínica de Enfermedades Infecciosas y Microbiología, Hospital Universitario de Valme, Instituto de Biomedicina de Sevilla (IBiS), Universidad de Sevilla, Avenida de Bellavista s/n, 41014, Sevilla, Spain; Unidad de Enfermedades Infecciosas, Hospital General Universitario Dr. Balmis, Instituto de Investigación Biomédica de Alicante (ISABIAL), Alicante, Spain

## Abstract

**Objectives:**

To investigate the role of previous antibiotic therapy in the risk of recurrence after a *Clostridioides difficile* infection (CDI) treated with vancomycin.

**Methods:**

Multicentre observational study. Patients with a CDI episode achieving clinical cure with oral vancomycin and followed up 8 weeks were included. Previous antibiotic exposure up to 90 days was collected. Multivariate analysis of predictors of recurrence adjusted by the propensity score (PS) of being previously treated with each non-CDI antibiotic was performed.

**Results:**

Two hundred and forty-one patients were included; 216 (90%) had received systemic antibiotics. Fifty-three patients (22%) had a CDI recurrence. Rates of recurrence were lower in those treated with piperacillin/tazobactam in the last month when compared with those not receiving piperacillin/tazobactam [3 (7%) versus 50 (25%); *P* = 0.01], whereas higher rates were seen in those treated with cephalosporins in the last month [26/87 (30%) versus 27/154 (17%); *P* = 0.03]. In multivariate analysis controlled by the inverse probability of treatment weighting by PS, receiving **≥**5 days of piperacillin/tazobactam in the last month as the last antibiotic regimen prior to CDI was independently associated with a lower risk of recurrence [adjusted OR (AOR) 0.13; 95% CI: 0.06–0.29; *P* < 0.0001] whereas exposure for **≥**5 days to cephalosporins (versus piperacillin/tazobactam) was associated with an increased risk (AOR 10.9; 95% CI: 4.4–27.1; *P* < 0.0001).

**Conclusions:**

Recent use of piperacillin/tazobactam might be associated with a lower risk of CDI recurrence, while recent use of cephalosporins might promote an increased risk. These findings should be considered when treating hospitalized patients.

## Introduction

*Clostridioides difficile* infection (CDI) is the leading cause of nosocomial infectious diarrhoea and one of the most prevalent healthcare associated infections.^1,2^ Antimicrobial exposure is one of most relevant individual factors associated with CDI development.^3^ Previous studies have shown that the risk of CDI is driven by the duration of antibiotic exposure but also by the specific class of antimicrobial agent received.^4–8^ In this sense, the risk of CDI seems to be higher after exposure to cephalosporins, carbapenems, quinolones and clindamycin when compared with other antibiotic regimens.^6–8^ Timing of exposure to antibiotics plays also an important role, as the higher risk seems to be concentrated in the first 90 days after antibiotic exposure.^5^ In this sense, the impact of antibiotics administered in the 60 days prior to admission seems to be more relevant than those administered during the index hospitalization in patients with healthcare associated CDI.^8^ In addition to direct patient exposure to antibiotics, the influence of antibiotic use on the risk of CDI may also operate at aggregate levels (i.e. ward or hospital use of antibiotics).^9^ Previous studies have shown that limiting hospital use of high-risk antibiotics is associated with a substantial decline in CDI.^10–12^ For the above-mentioned reasons, the implementation of antimicrobial stewardship programmes is one of the most important strategies to diminish the burden of CDI within hospitals.

Current first-line options for treatment of CDI, which include the use of fidaxomicin or vancomycin, achieve clinical cure after the end of therapy in at least 80% of patients.^13–15^ However, following discontinuation of CDI therapy, up to 15%–25% patients will experience a first recurrence of CDI,^13–15^ the risk of recurrence being progressively higher in subsequent recurrent episodes.^16,17^ Besides its clinical implications, the economic burden of recurrent episodes is substantial, mainly derived from increased length of hospital stays and readmissions.^18^ Importantly, cost-effectiveness models have shown that cost-effectiveness of CDI treatments are mostly affected by patient responses during their initial episodes.^19^ Thus, the optimization of CDI management at the initial episode with the aim of preventing recurrences is essential.

Recurrence of CDI has been attributed to a lack of recovery of intestinal microbiome diversity following CDI treatment and the persistence of *C. difficile* spores in the intestinal tract.^3^ Due to this, exposure to systemic non-CDI antibiotics has been associated not only with CDI development but also with an increased risk of recurrence.^20–22^ However, a recent meta-analysis found that there was insufficient evidence for a role of non-CDI antibiotics in recurrence.^23^ Additionally, there is scarce information on whether the risk of CDI recurrence varies depending on the specific class of antibiotics received previously to the initial CDI episode. As the impact of different systemic antibiotics on intestinal microbiome might differ, it is reasonable to speculate that the risk of recurrence could be also influenced by the specific class of antibiotic that induced CDI. A case–control study conducted in acute and long-term care Veteran Affairs facilities found that prior use of several antibiotic classes (including fluoroquinolones, cephalosporins and β-lactam/β-lactamase inhibitor combinations) was associated with an increased risk of CDI recurrence.^21^ However, a relevant limitation in that study was that the majority of patients were treated with oral metronidazole, which is no longer a first-line option for CDI therapy.^24,25^

Our objective was to investigate the role of previous systemic antibiotic therapy in the risk of recurrence after an initial CDI episode treated with vancomycin.

## Patients and methods

### Study design and patients

The ICD-ANCRAID-SEICV cohort (ClinicalTrials.gov ID: NCT04801862) is a prospective multicentric cohort recruiting all consecutive adult patients with a new diagnosis of CDI from 15 hospitals from Spain since October 2020. Besides, since 2018, all CDI cases diagnosed at the coordinator centre of the cohort (Hospital Universitario de Valme) have been prospectively followed up under a structured protocol of care, including data collection, and were used for the present analysis.

Patients were included in this study provided that they met the following inclusion criteria: (i) diagnosis of CDI; (ii) treatment of the CDI episode with a standard regimen of oral vancomycin; (iii) achieved clinical cure at the end of therapy with vancomycin; and (iv) completed a minimum follow-up of 8 weeks after the end of CDI treatment. Patients with a previous episode of CDI in the preceding 8 weeks and those who received adjuvant therapy with bezlotoxumab were excluded.

### Definitions and microbiological procedures

CDI diagnosis was established following the ECDC^26^ and ESCMID recommendations^24,27^ by the presence of diarrhoea and a positive laboratory assay for *C. difficile* toxin A and/or B in stools or a toxin-producing *C. difficile* organism detected in stools by means of a PCR method. According to ECDC criteria, a CDI episode was classified as healthcare-associated CDI (HA-CDI) if the onset of symptoms occurred on Day 3 or later following admission to a healthcare facility or within 4 weeks of discharge from a healthcare facility.^26^ Among HA-CDI cases, analyses were also performed considering only hospital-onset healthcare facility-associated (HO-HCFA) CDI cases as defined by IDSA.^25^ Clinical cure was defined as resolution of diarrhoea with maintenance of resolution for the duration of therapy and at least 48 h after the end of treatment.^24^ Recurrence was defined following ESCMID and IDSA criteria^24,25^ as the reappearance of symptoms within 8 weeks after a previous episode, provided the symptoms from the previous episode resolved after completion of initial treatment.

The algorithm employed for the microbiological diagnosis of CDI was based on the sequential qualitative detection of glutamate dehydrogenase and toxin A and B from *C. difficile* by immunochromatography (CerTest, BIOTEC S.L., Spain; *C. DIFF Quik Chek* Complete, TechLab, Blacksburg, VA, USA). We assessed discrepancies by detecting *C. difficile* toxin genes by real-time PCR (BD MAX Cdiff, Becton Dickinson Diagnostics, Canada; Xpert *C*.*difficile*/*Epi* test, Cepheid, Sunnyvale, CA, USA).

### Antibiotic exposure

Data from systemic antibiotic exposure up to 90 days previous to CDI diagnosis were collected. Antibiotic consumption prior to CDI diagnosis was collected by means of electronic clinical records, which are linked with in-hospital and community electronic prescriptions modules by a unique patient identifier. For each antibiotic, exposure was recorded as ‘yes’ or ‘no’ irrespective of treatment duration, provided that at least 48 h of treatment was completed. Patients with multiple courses of antibiotics were not excluded. Sensitivity analysis restricted to patients with a single course of antibiotic and those with exposure to antibiotics in the last 4 weeks before CDI diagnosis was also performed. A sensitivity analysis considering exposure to individual antibiotics of at least 5 days duration was also performed. Non-CDI antibiotics were defined as those not currently considered as treatment options for CDI in ESCMID clinical practice guidelines (oral vancomycin, metronidazole, fidaxomicin and/or tigecycline).

### Statistical analysis

The primary outcome variable of the study was the emergence of CDI recurrence. The possible association between the primary outcome and the following variables was assessed: age, sex, setting of infection, diabetes, active neoplasm, immunosuppression, chronic kidney disease, cirrhosis, bowel inflammatory disease, Charlson comorbidity index, major surgery in the previous 3 months, living in a long-term facility, hospitalization in the last year, treatment with proton pump inhibitors (PPIs) in the last 3 months, severity of the CDI episode according to IDSA criteria,^27^ number of bowel movements at diagnosis, persistence of diarrhoea at Day 5 of CDI treatment, exposure to non-CDI systemic antibiotics in the previous 90 days including the specific antimicrobial agent, concomitant antibiotic at CDI diagnosis and antibiotic use after CDI diagnosis.

Continuous variables were expressed as median (IQR) and categorical variables as frequencies (percentage). Continuous variables were compared by means of the Student’s *t*-test or the Mann–Whitney *U*-test, depending on whether a normal distribution was proven or not. Chi-squared and the Fisher tests were used for comparisons between categorical variables. Variables with a *P* value less than 0.2 on univariate analyses were entered in a logistic regression model, which also included age and sex. In order to control potential confounding bias due to differences between patients with previous exposure to different antibiotic classes showing univariate associations, propensity score (PS) analysis was performed. Thus, in the subcohort of patients who received systemic antibiotics prior to CDI, a PS for receiving a specific antibiotic agent was calculated using a non-parsimonious multivariable logistic regression model in which all potential predictors of receiving a specific antibiotic agent were included. The ability of the PS to predict the observed data was calculated by the area under the receiver operating characteristic curve (AUROC) with 95% CI. The performance of the PS was assessed by examining density plots. The PS was used in two ways: (i) as a covariate in a multivariable logistic analysis; and (ii) to form an inverse probability of treatment weighting (IPTW) subcohort. Differences were considered significant for *P* values ≤0.05. OR and the respective 95% CI were calculated. Data were analysed using IBM SPSS 24.0 version (IBM Corporation, Somers, NY, USA).

### Ethics

This study was designed and performed according to the Declaration of Helsinki and was approved by Hospital Universitario de Valme Ethics Committee (Ref. 1254-N-20).

## Results

### Features of study population

Seven hundred and forty-seven patients were diagnosed with CDI in the participant hospitals during the study period. Of those, 403 (54%) were excluded as they received initial treatment with regimens other than vancomycin. Figure S1, available as Supplementary data at *JAC-AMR* Online, summarizes the disposition of patients with the reasons for exclusion. Table 1 summarizes the main features of the 241 patients who finally comprised the study population. Five (2.1%) patients had suffered a prior CDI episode that was treated more than 8 weeks before the current episode.

**Table 1.** Features of the study population (*n* = 241)

Parameter	
Age (years), median (IQR)	75 (61–83)
<65	78 (32)
65–74	40 (17)
≥75	123 (51)
Female sex, *n* (%)	143 (59)
Living in a long-term facility, *n* (%)	11 (5)
Hospitalization in the last year, *n* (%)	125 (52)
Origin, *n* (%)^a^	
Community	65 (27)
Healthcare associated	173 (73)
HO-HCFA	116 (49)
Immunosuppression, *n* (%)	39 (16)
Chronic kidney disease, *n* (%)	62 (26)
Cirrhosis, *n* (%)	11 (5)
Charlson comorbidity index, median (IQR)^b^	5 (3–7)
≥3	183 (78)
Severity of CDI episode, *n* (%)^c^	
Non-severe	183 (78)
Severe	48 (21)
Fulminant	3 (1)
Systemic non-CDI antibiotics in previous 3 months, *n* (%)	216 (90)
Active systemic non-CDI antibiotics at diagnosis, *n* (%)	89 (37)
Use of PPIs in previous 3 months, *n* (%)	155 (64)

a Available in 238 patients.

b Available in 233 patients.

c Available in 234 patients.

Two-hundred and sixteen (90%) patients had received systemic non-CDI antibiotics in the previous 3 months (Table 1). Of those, 193 (80% of the study population) had received antibiotics in the last month. Eighty-nine (37%) patients were receiving antibiotics at the time of CDI diagnosis.

### Previous systemic non-CDI antibiotic exposure and risk of CDI recurrence

Fifty-three patients (22%) had a CDI recurrence during follow-up. Table 2 shows the risk of CDI recurrence according to previous systemic non-CDI antibiotic exposure. Previous antibiotic exposure showed a trend for an association with a higher risk of recurrence while active antibiotic treatment at the moment of CDI diagnosis was not associated with an increased risk (Table 2). When considering the specific antibiotic agent, previous exposure to cephalosporins at any time in the preceding 3 months was associated with an increased risk of recurrence when compared with patients with exposure to a different class of antibiotic (Table 2).

**Table 2.** Risk of CDI recurrence according to systemic non-CDI antibiotic exposure (*n* = 241)

Parameter	*n*/*N* (%) with recurrence	*P*
Previous antibiotic exposure		
No	4/25 (16)	0.1
Yes	49/216 (23)	
Concomitant antibiotic at CDI diagnosis		
No	33/127 (26)	0.9
Yes	20/89 (22)	
Aminopenicillins		
Yes	18/90 (20)	0.8
No but exposure to another antibiotic	31/126 (24)	
No exposure to antibiotics	4/25 (16)	
Piperacillin/tazobactam		
Yes	7/58 (12)	0.3
No but exposure to another antibiotic	42/158 (27)	
No exposure to antibiotics	4/25 (16)	
Cephalosporins		
Yes	31/107 (29)	0.01
No but exposure to another antibiotic	18/109 (16)	
No exposure to antibiotics	4/25 (16)	
Carbapenems		
Yes	11/43 (26)	0.2
No but exposure to another antibiotic	38/173 (22)	
No exposure to antibiotics	4/25 (16)	
Quinolones		
Yes	14/58 (24)	0.3
No but exposure to another antibiotic	35/158 (22)	
No exposure to antibiotics	4/25 (16)	
Clindamycin		
Yes	2/11 (18)	0.4
No but exposure to another antibiotic	47/205 (23)	
No exposure to antibiotics	4/25 (16)	
Fosfomycin		
Yes	10/25 (40)	0.02
No but exposure to another antibiotic	39/191 (20)	
No exposure to antibiotics	4/25 (16)	
Trimethoprim/sulfamethoxazole		
Yes	1/9 (11)	0.6
No but exposure to another antibiotic	48/207 (23)	
No exposure to antibiotics	4/25 (16)	

### Impact of class of antibiotic exposure in the last month on the risk of recurrence

**Figure 1.**
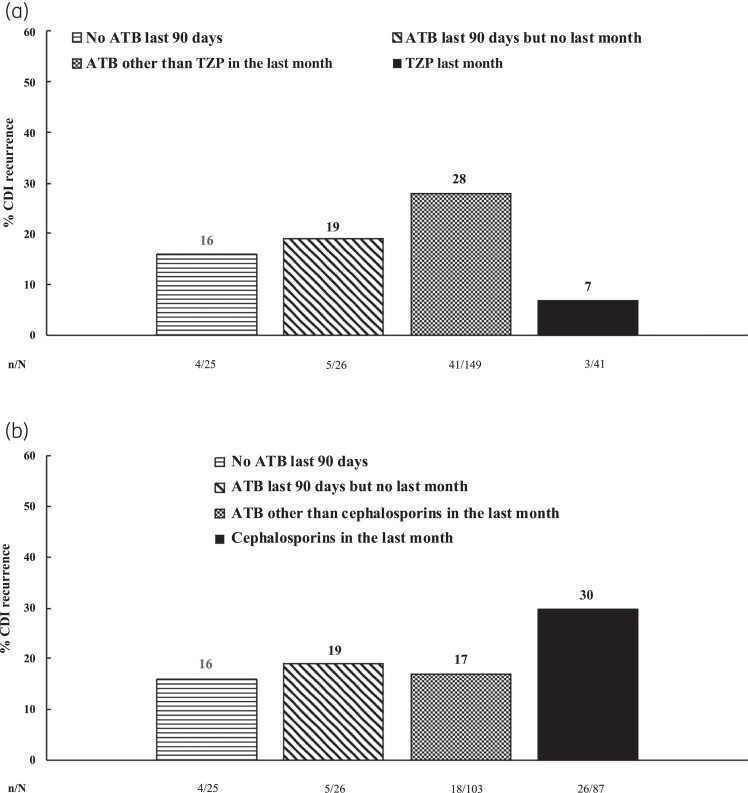
Risk of CDI recurrence according to previous exposure to systemic non-CDI antibiotics (ATB) (*n* = 244). TZP, piperacillin/tazobactam. (a) TZP. (b) Cephalosporins.

Forty-four out of 190 (23%) patients with exposure to antibiotics in the last 30 days and 4 out of 23 (17%) with antibiotic exposure in previous 3 months but not in the last 30 days developed a recurrence (*P* = 0.3). When considering the specific antibiotic agent, recent exposure to piperacillin/tazobactam was associated with a lower risk of recurrence. Thus, 3 out of 41 (7%) patients who received piperacillin/tazobactam in the last month had a recurrence, while this was observed in 50 (25%) patients without recent exposure to piperacillin/tazobactam (*P* = 0.01) (Table 3 and Figure 1a). On the contrary, use of cephalosporins in the last month was associated with a higher risk of recurrence when compared with those without recent exposure to cephalosporins [26/87 (30%) versus 27/154 (17%); *P* = 0.03] (Table 3 and Figure 1b).

**Table 3.** Predictors of CDI recurrence: univariate analysis (*n* = 241)

Factor	*n*/*N* (%) with recurrence	*P* bivariate
Age (years)		
<75	15/118 (13)	<0.001
³75	38/123 (31)	
Sex		
Male	26/98 (26)	0.15
Female	27/143 (19)	
Charlson comorbidity index		
<3	7/50 (14)	0.09
³3	46/183 (25)	
Cirrhosis		
No	49/228 (21)	0.2
Yes	4/11 (36)	
Chronic kidney disease		
No	40/179 (22)	0.8
Yes	13/62 (21)	
Bowel inflammatory disease		
No	51/234 (22)	0.6
Yes	2/7 (28)	
Immunosuppression		
No	44/202 (22)	0.8
Yes	9/39 (23)	
Living in a long-term facility		
No	48/230 (21)	0.07
Yes	5/11 (45)	
Hospitalization in the previous year		
No	21/116 (18)	0.2
Yes	32/125 (26)	
Origin^a^		
Community	13/65 (20)	0.9
Healthcare associated	15/57 (26)	
HO-HCFA	25/116 (22)	
Severity of CDI episode^b^		
Non-severe	39/183 (21)	0.7
Severe	12/48 (25)	
Fulminant	0/3 (0)	
Prior CDI episode		
No	51/236 (21)	0.3
Yes	2/5 (40)	
Use of PPIs		
No	17/86 (20)	0.5
Yes	36/155 (23)	
Exposure to CFP in last 30 days		
No	27/154 (17)	0.03
Yes	26/87 (30)	
Exposure to TZP in last 30 days		
No	50/200 (25)	0.01
Yes	3/41 (7)	
Last antibiotic regimen within 30 days prior to CDI diagnosis		
No exposure to antibiotics	9/48 (19)	0.02
CFP	19/56 (34)	
PIP/T	2/30 (7)	
Other	23/107 (21)	

CFP, cephalosporins; TZP, piperacillin/tazobactam.

a Available in 238 patients.

b Available in 234 patients.

We repeated the same analysis but considering only exposures to piperacillin/tazobactam or cephalosporins of at least 5 days duration during the last month. By this approach, recurrence was seen in 3 out of 38 (8%) patients with an exposure to piperacillin/tazobactam for ≥5 days in the last month whereas it was seen in 50 out of 203 (25%) of the remaining patients (*P* = 0.02). With respect to cephalosporins, 20 out of 66 (30%) patients with an exposure for ≥5 days within the last month had a recurrence whereas this occurred in 33 out of 175 (19%) remaining patients (*P* = 0.05).

Finally, we classified patients considering only the last course of antibiotic that the patients received in the last month prior to CDI diagnosis. Again, exposure to piperacillin/tazobactam was associated with a lower risk of recurrence (Table 3).

### Impact of class of antibiotic on the risk of recurrence in patients with a single course of antibiotics

In order to control for the possible confounding effect in patients receiving multiple courses of antibiotics, we further analysed the impact of the specific class of antibiotic on recurrence in 92 patients who underwent a single course of antibiotic in the last 3 months. A single course was defined if no change of antibiotic was made within the entire duration of antibiotic therapy. One (14%) out of seven patients treated with piperacillin/tazobactam had a recurrence, whereas this was seen in 17 (20%) patients treated with other options (*P* = 0.7). With respect to cephalosporins, 8 (31%) out of 26 patients had a recurrence whereas this occurred in 10 (15%) out of 66 treated with other options (*P* = 0.08). Recurrence rates were similar for those receiving quinolones and those receiving options other than quinolones [1/5 (20%) versus 16/87 (18%); *P* = 1.0].

### Antibiotic exposure after CDI diagnosis and risk of CDI recurrence

Exposure to antibiotics during the first 8 weeks after the end of CDI therapy and before recurrence could be assessed in 181 patients. Of these, 53 (22%) patients received antibiotics after CDI. Eight (28%) patients among those whose last antibiotic regimen prior to CDI was piperacillin/tazobactam received antibiotics during the first 8 weeks after CDI, whereas this occurred in 16 (35%) of those recently treated with cephalosporins and 16 (25%) of those previously treated with other options (*P* = 0.8). The rates of CDI recurrence among those who received antibiotics after CDI was 32% (17/53) versus 22% (28/128) in those not receiving antibiotics after CDI (*P* = 0.1).

### Independent predictors of CDI recurrence

A multivariate analysis including variables prior to CDI diagnosis to identify the independent predictors of CDI recurrence was performed. The independent predictors of CDI recurrence in the overall study population were age, male sex and recent exposure to cephalosporin-based antibiotic course (versus a piperacillin/tazobactam course) (model A, Table 4). We repeated this model including exposure to antibiotics within the 8 weeks after CDI and before recurrence. In this model, recent exposure to cephalosporins (versus piperacillin/tazobactam) was associated with a lower risk of recurrence [adjusted OR (AOR) 15.1; 95% CI: 1.7–133.2; *P* = 0.015] while exposure to antibiotics after CDI was not (AOR 1.5; 95% CI: 0.6–3.8; *P* = 0.3).

**Table 4.** Predictors of CDI recurrence: multivariate analysis

Factor	AOR	*P* multivariate
	(95% CI)	
Multivariate model without PS adjustment (*n* = 241)		
Age ≥ 75 years	3.2 (1.6–6.5)	0.001
Male sex	1.9 (1.01–3.7)	0.049
Charlson comorbidity index ≥ 3	1.1 (0.4–3.2)	0.7
Living in a long-term facility	2.1 (0.6–8.1)	0.2
Last antibiotic within 30 days prior to CDI		
TZP	Reference category	0.012
CFP	7.5 (1.5–36.1)	0.081
Other	3.9 (0.8–18.1)	0.099
No antibiotics	3.9 (0.7–20.5)	
Multivariate model with PS adjustment as a covariate (*n* = 193)		
Age ≥ 75 years	3.5 (1.5–8.2)	0.003
Male sex	2.9 (1.5–5.6)	0.001
Charlson comorbidity index ≥ 3	1.3 (0.4–4.03)	0.5
Living in a long-term facility	1.04 (0.3–3.3)	0.9
Last antibiotic within 30 days prior to CDI		
TZP	Reference category	<0.001
CFP	10.9 (4.4–27.1)	<0.001
Other	6.1 (2.6–14.1)	
PS for receiving TZP as the last antibiotic regimen within 30 days prior to CDI		
	0.7 (0.05–9.2)	0.7
Multivariate model with PS^b^ adjustment as a covariate (*n* = 193)		
Age ≥ 75 years	3.5 (1.5–8.1)	0.003
Male sex	2.9 (1.5–5.5)	0.001
Charlson comorbidity index ≥ 3	1.4 (0.4–4.1)	0.5
Living in a long-term facility	1.04 (0.3–3.3)	0.9
Last antibiotic within 30 days prior to CDI		
TZP	0.13 (0.06–0.29)	<0.001
Other	Reference category	
PS for receiving TZP as the last antibiotic regimen within 30 days prior to CDI		
	0.7 (0.06–9.5)	0.8
Multivariate model with IPTW by PS (*n* = 193)		
Age ≥ 75 years	3.6 (1.6–8.3)	0.002
Male sex	2.8 (1.5–5.4)	0.001
Charlson comorbidity index ≥ 3	1.3 (0.4–3.8)	0.6
Living in a long-term facility	1.1 (0.3–3.3)	0.8
Last antibiotic within 30 days prior to CDI		
TZP	Reference category	<0.0001
CFP	10.9 (4.4–27.1)	<0.0001
Other	6.1 (2.6–14.1)	
Multivariate model with IPTW by PS (*n* = 193)		
Age ≥ 75 years	3.6 (1.6–8.3)	0.002
Male sex	2.8 (1.5–5.4)	0.001
Charlson comorbidity index ≥ 3	1.3 (0.4–3.9)	0.5
Living in a long-term facility	1.1 (0.3–3.3)	0.9
Last antibiotic within 30 days prior to CDI		
TZP	0.13 (0.06–0.29)	<0.0001
Other	Reference category	

CFP, cephalosporins; TZP, piperacillin/tazobactam.

In order to control for indication bias, sensitivity PS-controlled analyses were performed in 193 patients receiving a non-CDI antibiotic course in the last month. For these analyses, non-CDI antibiotic exposure was initially categorized as: (i) receiving ≥5 days of piperacillin/tazobactam as the last antibiotic regimen; (ii) receiving cephalosporins for ≥5 days as the last antibiotic regimen; and (iii) other antibiotic regimens. First, a PS for receiving ≥5 days of piperacillin/tazobactam as the last antibiotic regimen in the last 30 days by logistic regression analysis was done. To calculate the PS, variables included in the logistic regression were age, sex, setting of infection, neoplasm, chronic kidney disease, Charlson comorbidity index, cirrhosis, inflammatory bowel disease, living in a long-term facility, immunosuppression, severity of the episode and previous CDI episode. The AUROC for PS was 0.78 (95% CI: 0.70–0.86). A multivariate logistic regression model including PS as a covariate was then performed (model B, Table 4). In this model, recent treatment with an antibiotic regimen other than piperacillin/tazobactam was associated with an increased risk of recurrence, the risk being higher with cephalosporins. This multivariate model was repeated but including recent antibiotic exposure to piperacillin/tazobactam as a dichotomic variable (model C, Table 4). In this model, recent exposure to piperacillin/tazobactam was associated with a lower risk of recurrence after adjustment for other covariates, including the PS. Multivariate logistic analysis with IPTW adjustment by PS for receiving piperacillin/tazobactam were also performed (models D and E, Table 4). By this approach, piperacillin/tazobactam was again associated with a lower risk of recurrence than exposure to cephalosporins.

## Discussion

Although the role of antibiotic exposure in the development of CDI is well established, its role in the emergence of CDI recurrence is still controversial. According to our results, recent use of piperacillin/tazobactam might be associated with a lower risk of recurrence, while recent use of cephalosporins might promote an increased risk. These findings should be considered when treating patients with healthcare-associated infections.

Antibiotics predispose to CDI via disruption of the gut microbiome. Previous evidence has confirmed that the risk of CDI development varies across different antibiotic classes,^6–8^ which might reflect different effects of antibiotics on the gut microbiome. Brown *et al.*^7^ found that the incidence of CDI was significantly higher after receiving a 7 day course of quinolones when compared with a 7 day course of amoxicillin for the same indication among nursing home residents in Ontario, Canada. In a retrospective study exploring the associations between antibiotic exposure and the risk of hospital-associated CDI cases, the greatest odds of CDI development were observed with the use of cephalosporins, piperacillin/tazobactam, carbapenems and quinolones prior to the admission in which CDI occurred, while the effect of antibiotic exposure during the index admission showed a lesser effect.^8^ Besides, in a clinical trial comparing the efficacy of fidaxomicin with that of vancomycin, clinical cure was less likely to be achieved in patients prescribed high-risk concomitant antibiotics, which was arbitrarily defined as carbapenems, cephalosporins, fluoroquinolones, clindamycin and extended-spectrum penicillins.^20^

A remarkable finding in our study was the lower rate of recurrence that was seen in those recently exposed to piperacillin/tazobactam when compared with patients treated with other classes of antibiotics, specially cephalosporins. As described for other agents, previous exposure to piperacillin/tazobactam had been associated with an increased risk of a first episode of CDI.^8^ By contrast, in a large retrospective cohort study conducted in the Veterans Health Administration, cefepime and meropenem were associated with an increased risk of CDI risk relative to piperacillin/tazobactam.^28^ Our results suggest that piperacillin/tazobactam could be less detrimental regarding CDI than other options, with several factors contributing to this. First, piperacillin/tazobactam has inhibitory activity against *C. difficile.* This was illustrated in a recent meta-analysis summarizing data on antimicrobial resistance of *C. difficile*, which found that the weighted pooled resistance of *C. difficile* for piperacillin/tazobactam was 0%, with only 8 (0.3%) out of 3041 isolates tested in 17 studies being resistant to this drug.^29^ Apart from its *in vitro* activity, piperacillin/tazobactam might achieve sufficient concentrations in the intestinal tract to inhibit *C. difficile* colonization during therapy. In line with this, in a study comparing the frequency of asymptomatic rectal carriage of toxigenic *C. difficile* among patients receiving piperacillin/tazobactam with that of patients receiving antibiotics lacking activity against *C. difficile*, such as cephalosporins or ciprofloxacin, patients receiving piperacillin/tazobactam were less likely to be asymptomatic carriers than those receiving non-inhibitory antibiotics or patients without active antibiotic therapy but with prior exposure within 90 days.^30^ This might be relevant, as the persistence of *C. difficile* spores in the gut can play a role in the recurrence of CDI. ^31^

Clarifying the role of the microbiome and its interactions with *C. difficile* within the gut is still a challenge. A relevant study recently published in *Nature* has confirmed that enterococci enhance *C. difficile* pathogenesis as they reshape the metabolic environment in the gut, favouring *C. difficile* colonization, toxin production and virulence.^32^ According to this new evidence, the differences in spectrum of activity against enterococci between piperacillin/tazobactam and cephalosporins might explain the different impact on CDI recurrence observed in our study. A reasonable hypothesis is that the disruption of gut microbiome induced by piperacillin/tazobactam might be less deleterious than that induced by cephalosporins or other options, as piperacillin/tazobactam might preserve against enterococci dominance in the microbiota, promoting a gut environment less prone to CDI recurrence. However, the effect of antibiotics on intestinal microbiota might differ not only due to the differences between the spectrum of antibacterial activity, but also according to the pre-treatment composition of the microbiota, the dose of antibiotics, the duration of the exposure or pharmacodynamics factors such as biliary excretion or faecal concentrations.^33^ Besides, it has been questioned if the measured activities of CDI antibiotics *in vitro* in a very well-defined environment against pure cultures of *C. difficile* represent what occurs *in vivo* in the intestinal tract with the presence of various other bacterial species and metabolites.^34^ Prospective analyses of faecal microbiota composition after CDI, adjusting for previous non-CDI antibiotic exposure, would be very informative to confirm or refute our observations.

Classically, discontinuation of the inciting antibiotic agent as soon as possible has been considered an important step in the management of CDI,^22^ as treatment with concomitant antibiotics other than those given to treat CDI has been associated with an increased risk of treatment failure and recurrent CDI.^20^ However, in many circumstances, antibiotic therapy is clinically indicated and therefore impossible to stop at CDI diagnosis. There is a lack of evidence regarding which antibiotic agents are preferable in the case that concomitant systemic antibiotic cannot be stopped. Although most experts advise selecting agents among those that are less frequently implicated in antibiotic-associated CDI,^22^ evidence supporting this practice is lacking. Moreover, a recent systematic review found that neither non-CDI antibiotic use prior to the primary CDI nor concomitant non-CDI antibiotics used during the primary CDI episode appeared to be convincing predictors for recurrent CDI.^23^ Our results raise the question of if it should be considered to give preference to piperacillin/tazobactam, when this is a suitable option, rather than other agents such as cephalosporins in those diagnosed with CDI and needing concomitant systemic antibiotics. Although the observational design of this study does not allow us to give a strong recommendation on this, our data should be taken into account in the complex process of daily antimicrobial stewardship decisions when caring for patients with healthcare-associated infections at high risk for CDI development.

Our study has some limitations. First, this is an observational study and the indication of previous systemic antibiotics were made by the caring physicians. Thus, observed associations between antibiotic classes and CDI recurrence might not imply causality as selection bias might have occurred. To avoid this, we performed multivariate analysis adjusted by the PS of receiving each specific drug. Second, patients with multiple antibiotic courses were not excluded, to assure that the real-life population with CDI was analysed. However, determining the role of each antibiotic agent in those receiving multiple ones is challenging and prone to confounding bias. To solve this, we performed several sensitivity analyses as those restricted to patients receiving a single antibiotic course and to antibiotics received in the last month. The selection of this interval was based on previous studies showing that the risk of CDI is highest during non-CDI antibiotic treatment and for up to 1 month after discontinuation.^5,35^ Besides, we also analysed the impact of the last antibiotic course. All the approaches show concordant results, suggesting that recent exposure to piperacillin/tazobactam when compared with cephalosporins was associated with a lower risk of recurrence. These findings were also seen when non-CDI antibiotic regimens were also evaluated taking into account treatment duration, as a dose–response association between community-acquired (CA)-CDI risk and antimicrobials has been previously suggested.^5^ Thus, we performed a sensitivity analysis restricted to patients with a recent exposure to piperacillin/tazobactam or cephalosporins of at least 5 days that yielded the same results. Finally, our findings only apply to patients undergoing treatment with a standard course of vancomycin as other treatment options were excluded, with the aim of avoiding confounding bias due to the lower rates of recurrence that are achieved with fidaxomicin or the use of bezlotoxumab. Future research should confirm our findings in patients with CDI treated with other options.

In summary, recent exposure to piperacillin/tazobactam prior to CDI diagnosis might be associated with lower rates of CDI recurrence when compared with the use of cephalosporins. While awaiting further confirmatory data, this information should be incorporated to guide clinical decisions from an antimicrobial stewardship perspective.

## Supplementary Material

dlad033_Supplementary_Data

## Supplementary data

Figure S1 is available as Supplementary data at *JAC-AMR* Online.
