# Correction to: Role of previous systemic antibiotic therapy on the probability of recurrence after an initial episode of *Clostridioides difficile* infection treated with vancomycin

**DOI:** 10.1093/jacamr/dlad121

**Published:** 2023-11-10

This is a correction to: Nicolás Merchante, Rocío Herrero, María Dolores Valverde-Fredet, Miguel Rodríguez-Fernández, Héctor Pinargote, Francisco J Martínez-Marcos, Concepción Gil-Anguita, María García-López, María Tasias Pitarch, Vicente Abril López De Medrano, Miguel Nicolás Navarrete Lorite, Cristina Gómez-Ayerbe, Eva León, Pilar González-De La Aleja, Ana Ruiz Castillo, Ana I Aller, Juan Carlos Rodríguez, Julia Ternero Fonseca, Juan E Corzo, Alberto Naranjo Pérez, Marta Trigo-Rodríguez, Esperanza Merino, Role of previous systemic antibiotic therapy on the probability of recurrence after an initial episode of *Clostridioides difficile* infection treated with vancomycin, *JAC-Antimicrobial Resistance*, Volume 5, Issue 2, April 2023, dlad033, https://doi.org/10.1093/jacamr/dlad033

In the originally published version of this manuscript, there was a typographical error in the name of author Héctor Pinargote.

This has been corrected.

