# Supplementary figures and images for: Role of previous systemic antibiotic therapy on the probability of recurrence after an initial episode of Clostridioides difficile infection treated with vancomycin
Source: JAC Antimicrob Resist. 2023 Mar 23;5(2):dlad033. doi: 10.1093/jacamr/dlad033 (PMC10035639; doi:10.1093/jacamr/dlad033)

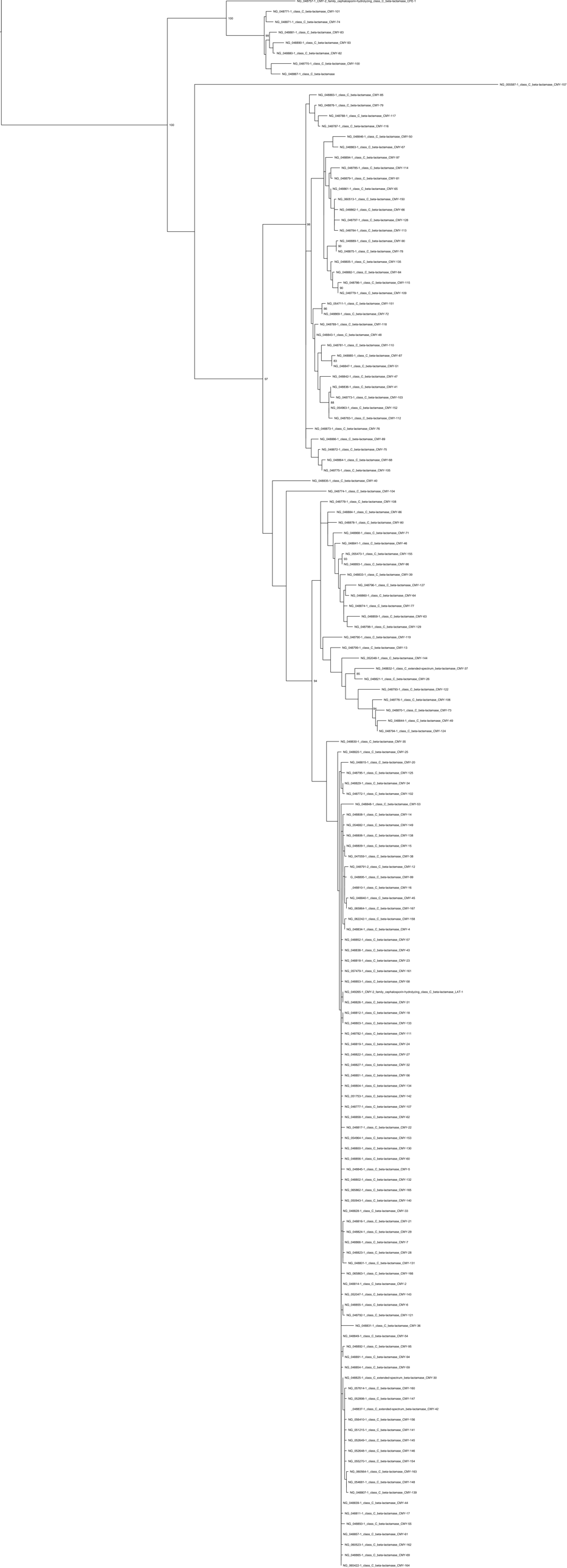

Supplement: dlad033_Supplementary_Data [file dlad033_supplementary_data.zip › Figure S1.pdf]
